# Targeting metabolic disorders by natural products

**DOI:** 10.1186/s40200-015-0184-8

**Published:** 2015-07-08

**Authors:** Ozra Tabatabaei-Malazy, Bagher Larijani, Mohammad Abdollahi

**Affiliations:** Diabetes Research Center, Endocrinology and Metabolism Clinical Sciences Institute, Tehran University of Medical Sciences, Tehran, Iran; Endocrinology and Metabolism Research Center, Endocrinology and Metabolism Clinical Sciences Institute, Tehran University of Medical Sciences, Tehran, Iran; Department of Toxicology and Pharmacology, Faculty of Pharmacy, and Pharmaceutical Sciences Research Center, Tehran University of Medical Sciences, Tehran, Iran

**Keywords:** Metabolic disorders, Oxidative stress, Epigenetic, Natural products

## Abstract

The most prevalent metabolic disorders are diabetes mellitus, obesity, dyslipidemia, osteoporosis and metabolic syndrome, which are developed when normal metabolic processes are disturbed. The most common pathophysiologies of the above disorders are oxidative stress, Nrf2 pathways, epigenetic, and change in miRNA expression. There is a challenge in the prevention and treatment of metabolic disorders due to severe adverse effects of some synthetic drugs, their high cost, lack of safety and poverty in some conditions, and insufficient accessibility for the general population in the world. With increasing interest in shifting from synthetic drugs to phytotherapy as an alternative treatment, there is still a gap in scientific evidences of plant-derived therapeutic benefits. One reason may be slow rate of translation of animal studies’ findings into human clinical trials. Since metabolic disorders are multifactorial, it seems that poly-herbal medications, or drug-herbal combination are needed for their treatment. However, further researches to determine the most effective plant-derived metabolites, and their cellular mechanism in order to set priorities for well-designed animal and clinical trials, and also more studies with strong scientific evidences such as systematic review and meta-analysis of controlled studies are needed.

## Introduction

Metabolism is the breaking down of food, in a highly regulated manner, into simpler components of proteins, carbohydrates, and fats. Metabolic disorders develop when normal metabolic processes are disturbed. The most prevalent metabolic disorders are diabetes mellitus, obesity, dyslipidemia, osteoporosis and metabolic syndrome. Many efforts have been made over the last decade to employ natural productsin drug development. More than two thirds of the active agents of drugs have relationship to natural sources [1]. Among 19 natural based drugs that have been approved for worldwide marketing between the years 2005–2010, 7 were classified as natural products, 10 as semi-synthetic natural products, and 2 as natural product-derived drugs. Some examples include Veregen ™ as a mixture of catechins derived from green tea against genital warts, Sativex® derived from Cannabis plant for pain relief, and Exenatide (Byetta®) isolated from *Heloderma suspectum* as adjunctive therapy in type 2 diabetes [2]. Among over the 100 natural product-derived compounds that were undergoing at different stages of clinical trials, 17 projects were about metabolic disorders in 2008 [3], and this figure is increasing according to registered trials in www.clinicaltrials.gov. In this review, it will be briefly discussed about the pathophysiology and pharmacology of currently available synthetic drugs, and also the role of natural products in the management of the above metabolic disorders.

### Diabetes mellitus

Diabetes mellitus is characterized by chronic hyperglycemia due to insulin resistance and defect in insulin secretion and/or insulin action caused by Langerhans islets’ β-cell failure [4]. Other primary defects responsible for development of diabetes are: increase in hepatic glucose production and decrease in peripheral glucose utilization [5]. This disease is one of the most important worldwide health problems that shows an increasing prevalence. According to the International Diabetes Federation’s (IDF) report there are approximately 381 million diabetic patients worldwide, a figure that expects to rise to 592 million by 2035 [6].

Diabetes mellitus has been classified into two forms; type 1 and type 2. Type 1 diabetes is caused by autoimmune destruction of β-cells secondary to environmental triggers such as toxins and viruses. Therefore treatment of type 1 diabetes depends on exogenous insulin. This type of diabetes accounts for about 10 % of all diabetic patients and more frequently seen in children and young adults [6]. Type 2 diabetes is more prevalent than type 1 and considered as a heterogeneous disease.

#### Pathophysiology of diabetes mellitus

Several studies have demonstrated that oxidative stress has an important role in pathogenesis of diabetes and its complications [7, 8]. Generally, oxidative stress is defined as an imbalance between reactive oxygen species (ROS) production and enzymatic or non-enzymatic antioxidants’ capacity. ROS includs: free radicals such as superoxide (^●^O_2_^−^), hydroxyl (^●^OH), peroxyl (^●^RO_2_), hydroperoxyl (^●^HRO_2_^−^), and non-radical species such as hydrogen peroxide (H_2_O_2_) [9]. Superoxide dismutase (SOD), glutathione reductase, vitamins A, C and E, carotenoids, glutathione and trace elements [10] are some examples of antioxidants.

ROS are not always bad. Oxidative stress happens when an imbalance between ROS and natural antioxidant defense in the body develops. This imbalance can be observed in some chronic disorders such as diabetes mellitus [7]. ROS can stimulate the oxidation of low density lipoprotein cholesterol (LDL-C), that after taking up by scavenger receptors in macrophages, results in foam cell formation and atherosclerotic plaques [11]. The free radicals and non-radical species can activate several damaging pathways that all of them have important roles in the development of diabetes’ complications. Some of these pathways are: polyol pathway, hexosamine pathway, mitochondrial respiratory chain, accelerated advanced glycation end products (AGEs) formation, activation of protein kinase C (PKC), stimulation of stress-related signaling mechanisms such as Nuclear factor κB (NF-κB), p38 mitogen-activated protein kinase (p38-MAPK), and Janus kinase-signal transducer and activator of transcription (STAT-JAK) [11, 12]. Activation of these pathways and mechanisms can result in endothelial dysfunction, cell apoptosis, pathological angiogenesis, peroxidation of membrane lipids, autoxidation of glucose (and AGEs formation), islet amyloid deposition, and β-cell mass and function failure [13–16].

Loss or dysfunction of pancreatic islet cells is involved in pathogenesis of both types of diabetes. There are some evidences that epigenetic factors may contribute to diabetes [17–19]. Epigenetic is defined as stable alterations in gene expression but not at the DNA sequence, in response to environmental stimuli and nutrients [20]. On the other word, a combination of genetic and/or epigenetic changes engendered during the period of oxidative stress, can result in an altered cellular “memory” and predisposition to diabetes [21]. Recently microRNA (miRNA) is known as an essential factor for normal pancreas development. The miRNA is a small noncoding RNA that can modulate gene expression at post-transcriptional level [22]. Alteration in expression of a number of different miRNA has been observed during diabetes development [23, 24]. DNA methylation and increases in miRNA expression can lead to reduced transcriptional activity of key β-cell genes (Pdx1 and insulin). Combination of enhanced ROS with decreased Pdx1 protein and insulin can result in intensification of apoptosis.

It is known that transcription factor Nuclear factor-erythroid 2-related factor 2 (Nrf2), in association with its negative regulator, Kelch-like ECH-associated protein 1 (Keap1) are one of the most important cellular defense mechanisms against oxidative stress [25]. In addition, employment of NADPH oxidase (Nox family) inhibitors such as GKT136901 and GKT137831 may be a useful strategy to reduce ROS-induced damage [26].

#### Currently available anti-diabetic synthetic drugs

Current validated available anti-diabetic drugs are: Metformin, sulfonylurea, and insulin. Less validated new drugs includes thiazolidinediones, glucagon-like peptide-1 (GLP-1) receptor agonists, α-glucosidase inhibitors, Pramlintide and dipeptidyl peptidase-4 (DPP-4) inhibitors. The mechanism of action of each drug group for lowering the blood sugar is described below [27, 28].

### Biguanides (Metformin)

Metformin is the only biguanide that is available in most regions of the world. Its mechanism for lowering the glucose level is based on reduction of hepatic glucose output and enhancement of insulin sensitivity in skeletal muscle and adipocytes. Metformin monotherapy is expected to decrease HbA1C by 1.0–2.0 % [28]. The major non-glycemic effect of Metformin is either weight stability or modest weight loss. There are some evidences that Metformin has antioxidant properties. Reported antioxidant properties of Metformin are: decreasing the xanthin oxidase and lipid peroxidase activity, increasing the enzymatic antioxidants activity, chelating metal ions such as copper and iron, scavenging oxygenated free radicals generations, decreasing ROS level by activating different pathways such as AMP-protein kinase (AMPK)-mediated signaling, inhibition of AGEs formation, decreasing β-cell apoptosis, and also decreasing the production of tumor necrosis factor-α (TNF-α) [7, 29, 30]. Improvement of some markers of endothelial function in patients with type 2 diabetes has been reported by Metformin usage [31].

### Sulphonylurea

This group can reduce the glucose level by increasing the insulin secretion. Their major adverse effect is hypoglycemia that sometimes is accompanied by coma or seizure especially in elderly patients. This group can reduce the HbA1C level by 1.0–2.0 % [28]. Glibenclamide (glyburide), as a member of this group, displays antioxidant properties by scavenging free radicals and decreasing ROS levels by activating AMPK-mediated signaling [30, 32]. Glipizide is another member with antioxidant properties which is able to scavenge free radicals and restore antioxidant activities in various tissues of diabetic rats [33].

### Insulin

Of all the diabetes medications, insulin is the most effective in lowering the glycemia. It is expected to have a decrease by 1.5–3.5 % in HbA1C level with insulin monotherapy. Beneficial effects of insulin are shown on triglycerides and high density lipoprotein cholesterol (HDL-C) levels. Insulin therapy is associated with weight gain and hypoglycemia. Compared to Neutral Protamine Hagedorn (NPH) and Regular insulin, insulin Lispro can decrease the risk of hypoglycemia [28].

### Thiazolidinediones

Thiazolidinediones (TZDs or Glitazones) increase the sensitivity of muscle, fat and liver to endogenous and exogenous insulin. Their most common adverse effects are weight gain and fluid retention manifested as peripheral edema or heart failure. This group can reduce the HbA1C level by 0.5–1.4 % [28]. Rosiglitazone and Pioglitazone are examples of this group. An improvement in endothelial function in non-diabetics with hypertension or hypercholesterolemia has been reported by Pioglitazone [34]. Rosiglitazone is reported to decrease the inflammatory markers levels in hypertensive diabetic patients [35]. Reduction of β-cell apoptosis is another antioxidative property of this group [29]. However several meta-analysis have showed a 30–40 % increase in relative risk of myocardial infarction by Rosiglitazone [36, 37]. TZDs protect β-cells by reducing demand on the pancreas directly via peroxisome proliferator-activated receptor γ (PPARγ). TZDs can improve β-cell function by: reducing the risk of glucose toxicity and lipotoxicity, suppression of TNF-α, enhancement and proliferation of β-cell mass, reduction of β-cell apoptosis, reduction of islet amyloid, suppression of NF-κB activation, improvement of insulin secretion pattern, reduction of insulin resistance, reduction of thiobarbituric acid reactants (TBARs) levels and enhancement of catalase (CAT) activity [10, 29, 38].

### Glinides

Repaglinide and Nateglinideare examples of this group that can stimulate insulin secretion by binding to a different site of the sulphonylurea receptor. Due to short circulating half-life, they should be administrated frequently. A 0.5–1.5 % decrease in HbA1C is expected with monotherapy of glinide [28]. The major adverse effect of glinides is the risk of weight gain. It was shown that Repaglinide consumption can result in protein peroxidation inhibition, by up-regulation of glutathione reductase, and glutathione levels in diabetic rabbits [39].

### α-Glucosidase inhibitors

This group contains Acarbose, Voglibose and Miglitol. They can inhibit a family of membrane-bound enzymes in the brush-border of the small intestine, prevent the digestion of complex carbohydrates and retard glucose absorption. Their major adverse effect is poor gastrointestinal tolerance. Although they reduce the NF-κB activity, their antioxidant and anti-inflammatory effects are not yet completely approved [40].

### Incretin-based therapies

Incretin hormones are consisted of glucose dependent insulinotropic polypeptide (GIP) and GLP-1. They are released from endocrine cells in small intestine in response to high glucose levels to stimulate insulin secretion. In addition to central effects such as effects on gastric emptying or satiety, GLP-1 can reduce the secretion of glucagon and inhibit the conversion of glycogen to glucose by the liver. In contrast GIP can directly stimulate the glucagon secretion without having any central effects. A defect in GLP-1 secretion as well as resistance to GIP have been shown in type 2 diabetic patients. GLP-1 receptor agonists can increase insulin release and inhibit glucagon secretion with a low risk of hypoglycemia [41]. The native GLP-1 and GIP are inactivated rapidly by DPP-4. So, therapeutic strategies have prompted to develop DDP-4 resistant GLP-1 analogues such as Exenatide, Liraglutide as well as DPP-4 inhibitors; Sitagliptin, Vildagliptin, and Saxagliptin [42].

Exenatide is a synthetic form of the naturally occurring exendin-4 that binds avidly to GLP-1 receptor on β-cells and augments glucose-mediated insulin secretion, suppresses glucagon secretion and decreases gastric motility. A systematic review and meta-analysis study showed that Exenatide had beneficial effects not only on blood glucose but also on blood pressure and lipid profiles of diabetic patients [43]. However, itssuperiority over insulin therapy was not confirmed in another meta-analysis [44]. Its major adverse effect including gastrointestinal disturbances; nausea, vomiting, and diarrheathat are partially tolerabale [43]. Liraglutide is a long-acting GLP-1 analogue that has efficacy similar to that of Exenatide but with a better efficacy on glycemic status and fewer gastrointestinal side effects. The preference for long-acting GLP-1 agonists has been led to the development of modified dosage form of Exenatide, Albiglutide® and Taspoglutide®. A 0.5–1.0 % decrease in HbA1C with monotherapy of GLP-1 agonists is expected. More recently, the US Food and Drug Administration (FDA) has issued following warnings: pancreatitis with Exenatide or Sitagliptin® [28] and time and dose-dependent medullary thyroid carcinoma in rats and mice with Liraglutide. The main antioxidative effects of GLP-1 are: antiapoptotic action of islet cells and increase in proliferation, neogenesis and mass of β-cells [29]. DPP-4 inhibitors prolong the effects of GLP-1 and GIP, increase glucose-mediated insulin secretion, suppress glucagon secretion, and enhance β-cell function with a neutral effect on weight. A 0.6–0.9 % reduction in HbA1C level with monotherapy of DPP-4 inhibitors has been shown by clinical studies. Both GLP-1 analogues and DPP-4 inhibitors have indirect beneficial effects on lipogenesis, insulin resistance, and thermogenesis [41]. Antiapoptotic effect and increase in β-cell mass have been reported for DDP-4 [29].

### Amylin agonists

Amlin is a neuroendocrine hormone that is co-secreted with insulin in response to meals. Amylin-mediated activity results in suppression of postprandial glucose excursions, although its response is impaired in diabetes. Pramlintide is a synthetic analogue of amylin that is approved only as adjunctive therapy with insulin. It produces 0.5–0.7 % reduction in HbA1C with a high rate of gastrointestinal side effects and weight loss. The risk of hypoglycemia is high in adjunctive therapy of Pramlintide with insulin. Adjustment of insulin dosage is critical to reduce the risk of hypoglycemia [45].

#### New therapeutic agents

Edaravone is a member of this group that is not only a potent free radical scavengerand inhibitor of lipid peroxidation, but also has anti-apoptotic, anti-necrotic, and anti-cytokine effects [46]. Other members of this group are Benfotiamine that prevents oxidative DNA damage and poly (ADP-ribose) polymerase (PARP) inhibitors that reduce NF-κB activity, inhibit iNOS expression, and induce hydroxyl radical and hydrogen peroxide scavenging activities [30]. Recently, it was shown that interleukin-1 receptor antagonists (IL-1Ra) such as Anakinra (Kineret®) can reduce hyperglycemia and improve insulin sensitivity and pancreatic islets’ function. However, its clinical trial is still ongoing [47]. Other agents that can be considered as targets are: fibroblast growth factor 21 (FGF21), epoxide hydrolase (EPHX2) and ceramide synthesis inhibitors. Activators of ceramide degradation and sphingosine modulators (SK1, SK2), and P66Shc/RAGE signaling as target of hyperglycemia memory in vessels are new compounds that may be effectively protected against oxidative stress [30].

### New anti-diabetic indication of approved drugs

One therapeutic strategy to modulate oxidative stress in diabetes is supplementation therapy with antioxidative vitamins. In a meta-analysis, vitamin E supplement did not show any influence on glycemic control and fasting blood sugar inwhile another meta-analysis showed beneficial effects of vitamin C consumption on fasting blood sugar [48, 49].

Another therapeutic strategy to modulate oxidative stress in diabetes is to exploit the pleiotropic properties of drugs directed primarily at other targets and thus acting as indirect antioxidants. Some examples of this group are: statins, angiotensin-converting enzyme inhibitors (ACEI), β-blockers, Colesevelam, Bromocriptine, and Dapagliflozin. ACEI have shown to produce some antioxidative activity by inhibiting the NADPH oxidase and NO production, increasing the SOD activity and decreasing the malondialdehyde level [7, 30]. The antioxidative effects of statins as coenzyme A reductase inhibitors includes: suppression of NADPH oxidase expression, induction of antioxidant enzymes, reduction of circulating oxidized LDL-C and biomarkers of oxidatation F2-isoprostane and nitrotyrosine [50]. Nebivolol and Carvedilol belong to β-blocker group that block β-adernergic receptors and enhane vasodilation via block α-adernergic receptor. Both of them can inhibit activity and expression of NADPH oxidase, increase NO production, inhibit lipid peroxidation, and scavenge free radicals [7, 51, 52]. Colesevelam as a bile acid sequestrant for treatment of hyperlipidemia is now thought to delay or alert absorption of glucose from the intestines with a 0.41 % reduction in HbA1C when used alone or in combination with oral hypoglycemic agents [53]. Bromocriptine is a dopamine-2 receptor agonist that is shown to decrease HbA1C by 0.6 and 1.2 % when used as monotherapy or in combination with insulin or a sulphonylurea, respectively. Canagliflozin is a sodium-glucose transporter 2 (SGLT2) that prevents renal glucose reabsorption and reduces serum glucose level by increasing its urinary excretion. A 0.37–1.16 % reduction in HbA1C lvels is shown with SGLT2 employment. Its consumption is associated with increase in the risk of genital mycotic infections, urinary frequency, elevation of blood urea nitrogen and creatinine, diarrhea and fatigue [54]. Other drugs that could be considered in this section are Allopurinol, Pentoxifylline, Orlistat, anti platelet drugs (Aspirin, Clopidogrel), L-arginine, phosphodiesterase inhibitors (such as Theophylline), and pentaerythritol tetranitrate [7, 40, 55–57].

Despite the clear benefits of glycemic control and the availability of newer and potentially more effective drugs for management of diabetes, the number of patients with poor glycemic control has not been decreased over the past 10 years. These results may indicate that newer therapeutic agents are being underutilized or prescribed too late. We need to focus on newer agents that could affect the natural course of diabetes and underlying pathology [5, 27, 58]. However, we should not forget that the new medications have higher cost and lower availability in the world.

#### Natural products in the management of diabetes

In addition to the long history of natural products’ consumption as a medication [59], the popularity and use of alternative therapies are increasing dramatically. A telephone survey in 2002 in the US demonstrated that 18.8 % of American adults weekly use a dietary natural product supplement; herbal or other natural product. This figure was reported to be 14.2 % in 1998–1999 [60]. Since synthetic antidiabetic drugs have adverse effects such as severe hypoglycemia, and in some conditions they lack safety, considering alternative medicines for the management of diabetes is necessary. Alternative medicationsshould generally be safer, cheaper and be more easily available [61]. Based on the World Health Organization (WHO) report, 65–80 % of the world’s population are living in developing countries. This population needs to have access to herbal medicines because they have no access to modern medicine. Moreoveralternative medicine appears to be more conformable with patients’ beliefs and values [62]. Herbal medicine as one of the most popular alternative medicines has key role in the treatment of diabetes in Eastern and some of Western countries; German, France, Italy and the US [63–65]. During recent decades, modern medicine has achieved explosive developments, but plants are still a cone stone of healthcare and medical prescriptions. Some examples are Ruboxistaurin as a PKC inhibitor against microvascular complications of diabetes, Trodusquemine isolated from *Squalus acanthias* for treatment of type 2 diabetes, and Pyridoxamine (Pyridorin™) against diabetic nephropathy [2].

The large number of natural products-derived compounds that are currently in the ongoing stages of clinical trials, indicates that natural products are still a viable source for discovery new drug . Evaluation of medicinalplant’s efficacy for treatment of disorders such as diabetes has been recommended by WHO [66]. Nowadays, herbal products are used for prevention, mitigation and treatment of some diseases, however, some of them are reported to be unsafe and only a few has been evaluated adequately by modern tests [66–70]. There are some evidences on the safety and efficacy of herbal medicines, although they are not strong enough and we need to have more systematic review and meta-analysis. Meta-analysis of nine randomized, placebo-controlled trials showed that *Ipomoea batatas, Silybum marianum* and *Trigonella foenum-graecum* can significantly improve glycemic status [71]. Most of the studies on herbal medicine have shown antioxidative activity as the main mechanism [67, 72]. In this sectionevidences of the effects of natural products by focusing on antioxidative effects will be discussed.

### Polyphenols

The main character of the plant phenolics is aromatic natural products. This group consists of anthocyanins and flavonoids. Flavonoids are classified into 5 subgroups: flavonols, flavans, flavanols, flavones, and isoflavones. The plants of this group are well known as natural antioxidants that can scavenge free radicals, activate antioxidant enzymes, chelating metal, increase resistance of cell to oxidative stress and reduce adverse effects of ROS and reactive nitrogen species (RNS) [57, 73]. Modulation of miRNAhas been shown for quercetin, resveratrol, epigallocatechin-3-gallate (EGCG), Ellagitannin, and phenolic extract of *Hibiscussabdariffa* [22]. Recently, a systematic review conducted to assess randomized controlled clinical trials whereby resveratrol was used as an adjunct to pharmaceutical interventions in T2DM. Results showed the statistically significant positive effects of resveratrol versus placebo/control for systolic blood pressure, HbA1C, and creatinine, but not for fasting glucose, homeostatic model assessment of insulin resistance (HOMA index), diastolic blood pressure, insulin, and lipid profiles. This study suggested that resveratrol could be a candidate for adjunction therapy for pharmacological management of type 2 diabetes [74].

*Rhizoma Coptidis, Curcuma Longa, Pueraria Lobata*, and quercetin are some examples of this group. Quercetin is an important flavonoid that can be found in various foods such as citrus fruits, apples, onions, and tea. It can increase insulin secretion by enhancing hepatic glucokinase activity, or changing intracellular calcium concentration. Quercetin in combination with other polyphenols such as apigenin and luteolin can increase the viability of β-cells, insulin secretion, resistance to cytokine-induced cytotoxicity, decrease NO synthase and NF-κB activation [72, 75, 76].

Soybean is the main source of isoflavones in the human diet that consists of three principal isoflavones: genistein, daidzein and glycetein. Out of them, genistein is the most abundant isoflavones in soybean. Genistein can increase glucose-stimulated insulin secretion of the pancreas, and increase peripheral glucose uptake. It can protect β-cell against cytokine-induced toxicity, inhibit NO production and inhibit extracellular signal-related kinase-1/2(ERK-1/2), JAK/STAT pathway [77]. Other antioxidative effects of genistein are: chelating metals, reducing TNF-α and pro-inflammatory cytokine production, decreasing ROS, suppressing NF-κB binding activity, and restoring antioxidant enzymes [78].

The polyphenol curcumin as diferuloylmethane is the active ingredient of the dietary spice turmeric. It is derived from the plant *Curcuma longa* that has been used for so many years as a traditional medicine in China and India. Some of its antioxidative effects are inhibition of pro-inflammatory cytokines and lipid peroxidation, scavenging free radicals, inhibition of NF-κB activity, reduction of adhesion molecules and antiapoptotic proteins. All of 50 clinical trials conducted by usage curcuminhave supported that curcumin might be helpful in adjuvant therapy of type 2 diabetes [79–82].

### Nrf2 activators

Several activators of Nrf2 have been identified to combat oxidative stress-mediated disorders. The most well-known Nrf2 activators used in complementary medicine include sulforaphane, resveratrol, curcumin, cinnamic aldehyde, pterostilbene, a series of triterpenoids, and its derivatives [25]. Two examples of such activators that are currently being used in clinical trials are the synthetic triterpenoid bardoxolone methyl, or natural isothiocyanate sulforaphane, which is extracted from broccoli sprouts [83]. However, the early termination of a phase 3 clinical study by using bardoxolone methyl in patients with chronic kidney disease and type 2 diabetes showed that researchers should be more cautions inmanipulation of this pathway.

### Herbal phosphodiesterase inhibitors (PDEIs)

The PDEIs can inhibit phosphodiesterase and increase cAMP and cGMP. This group includes: flavonoids (*Sophora flavescns*, *Caesalpinia sappan*, *Ginkgo biloba*, *Berchemia floribunda*), alkaloids (*Picrasma quassiodes)*, saponins (*Periandra dulcis*), lignans (*Forsythia suspense*), coumarins (*Angelica pubescens*), and essential oils and resins (*Haplopappus rigidus*) [84]. It was reported that anti-inflammatory and antioxidant effects of PDEIs are related to prevention of induction oxidative stress and lipid peroxidation, elevation of antioxidant capacity, and improvement of function of isolated islet cells.

### α-lipoic acid

The α-lipoic acid, as a dithiol compound is derived from octanoic acid. Its antioxidative effects include: scavenging free radicals, chelation metal ions, and recycling antioxidant. Improvement of diabetic polyneuropathy, neural blood flow, nerve conduction and retinopathy in experimental models have been reported by consumption of this agent [7].

### Melatonin

The pineal endogenous hormone, melatonin (5-methoxy-n-acetyl-tryptamine) can modulate immune system activity, limit tumorigenesis, and inhibit oxidative stress. It is a scavenger of free radicals and protects against free radical-induced damage to DNA, proteins and membranes. Other effects are increase the CAT activity, decrease the hepatic GSH-peroxidase activity, decrease the lipid peroxidation. Significant beneficial effects on diabetic nephropathy and neuropathy in experimental studies have been shown [7].

#### Combination therapy in management of diabetes

Combination therapy is defined as consumption of eithet a combined synthetic drugs with polyherbal mixture or synthetic drugs with phytochemical compounds. Anti-diabetic plants contain thousands of components that only few of them have therapeutic effects. The synergistic effects of some herbal mixture has been reported [81, 85]. For example, the synergistic effects have been shown for combination of cinnamic acid derivatives with Metformin or TZDs which results in increased glucose uptake by L6 myotubes and 3 T3-adipocytes as well as increased in expression genes involved in the insulin cascade [86].

Although, it is believed that polyherbal formulation has synergistic effects, there are a few reports in their usefulness in diabetics [87]. However, due to the complex pathogenesisof diabetes, we will require multi-models for management of diabetes. Evaluation of synergistic effects of combined various plants with different anti-diabetic drugs could serve as one of these models [81]. Some examples of these mixture modes are IMOD® (*Rosa canina, Urtica dioica* and *Tanacetum vulgare* plus selenium and urea in a pulsed electromagnetic field), HAL (*Momordica charantia*, *Trigonella foenum-graecum*, *Withania somnifera*), DIA-2 (*Allium sativum*, *Lagerstroemiaspeciosa*) [87, 88]. The majority of studies employed polyherbal formulation in animal models of diabetes and human reported the antioxidative effects. However IMOD® showed an ameliorating effect on oxidative as well as immunological distresses of type-1diabetes [89].

### Obesity

Obesity is defined as an excess and abnormal accumulation of adipose tissue. It represents one of the most enormous global health problems with increasing prevalence that affects both genders and every ethnicity at any ages. According to a systematic review published in 2014, The worldwide prevalence of overweight was 36.9 % in men and 38 % in women in 2013 that was increased compared to 1980. According to this study 671 million obese individuals live in the world [90]. The accepted definition of overweight and obesity is based on body mass index (BMI) which presented in figures from 25.0 to 29.9 kg/m^2^ and ≥30.0 kg/m^2^, respectively. The genetic predisposition, environmental and behavioral factors act in concert to develop obesity. Obesity is associated with many disorders; diabetes mellitus, metabolic syndrome, cardiovascular risk factors (hypertension, dyslipidemia), some forms of cancers (breast, endometrial), pulmonary diseases (asthma, obstructive sleep apnea), gastrointestinal diseases, nonalcoholic fatty liver disease, and other disorders such as gout, osteoarthritis, etc. [91]. The obesity epidemic is also opposing enormous costs on health system due to increase in the risk of morbidity and mortality.

#### Pathophysiology of obesity

Generally, obesity is caused by an imbalance between food intake and energy expenditure and regulated by a complex physiological system from central regulation by the brain to peripheral signals. Adipose tissue is in two types; white adipose tissue (WAT) that presents the predominant type of adipose tissue and brown adipose tissue (BAT). BAT is mainly involved in thermogenesis, while WAT serves diverse functions. Adipose tissue, as an endocrine organ, has some secretory products with physiological functions including effect on energy homeostasis and metabolism, lipid and bone metabolism, steroid hormone conversation, sexual maturation, coagulation and fibrinolysis, hematopoiesis, angiogenesis, vasoconstriction/ vasorelaxation, modulation of the immune system, and kidney function [92].

The basic pathophysiology of the obesity is an enlargement of fat cells. Obese fat cells have increased production of inflammatory adipokines (TNF-α, interleukins 6 & 18), plasminogen activator inhibitor-1 (PAI-1), angiopoietin-like protein 2 (Angptl 2), vascular cell adhesion molecule-1 (VCAM-1) and monocyte chemoattractant protein-1 (MCP-1) that all of them could promote obesity-induced metabolic disorders [93, 94]. A protective effect against obesity-induced metabolic dysfunction has been reported for adiponectin. Adiponectin can activate AMPK in skeletal muscle and liver, increase insulin sensitivity, modulate inflammation, inhibit transformation of macrophage to foam cell via suppressing the expression of scavenger receptor-A (SR-A), reduce expression of VCAM-1 and interleukin-8 via inhibition of NF-κB, reduce inducible nitric oxide synthase (iNOS), stimulate production of interleukin-10, and promote ischemia-induced revascularization through stimulation of expression of cyclooxygenase-2 (COX-2) and prostaglandin I_2_ (PGI_2_) [94]. Obese individuals have very high levels of leptin, but serum level of adiponectin paradoxically has been decreased in their adipocytes. Other adipocytokines are: secreted frizzled-related protein 5 (Sfrp-5) and adipolin. It was shown that Sfrp-5 deficiency exacerbates fat inflammation and insulin resistance through enhancement of c-Jun N-terminal kinase (JNK1) activation in fat tissue. Adipolin can suppress pro-inflammatory mediators and promote insulin sensitivity [94].

It has been shown that serum level of adiponectin and systemic oxidative stress are inversely linked together. On the other hand, this inverse association was found between adiponectin level and risk of type 2 diabetes in a systematic review [95]. In contrast to adipocytokines, oxidative stress is increased during obesity and promotes obesity-induced metabolic dysfunction by increasing ROS in adipose tissues. ROS suppresses adiponectin mRNA expression and increases the mRNA expression of pro-inflammatory adipocytokines (interleukin-6 and MCP-1). On the other hand, a common role for PPARγ, in regulation of adiponectin and antioxidant enzymes, has been shown. Thus adiponectin can protect against oxidative stress-induced damage. Moreover antioxidative agents can restore adiponectin production [96]. Gut microbiota has a direct role in obesity development by regulating weight, and indirectly by acting through the gastrointestinal (GI) peptides. Similar to diabetes, obesity predisposition and weight loss outcomes are repeatedly associated with changes in epigenetic patterns secondary to nutritional and non-nutritional factors, such as hyperglycemia, inflammation, hypoxia and oxidative stress [97].

Nrf2 is a transcription factor that responds to exogenous and endogenous oxidative stress by binding to the antioxidant response element (ARE). Recently it is demonstrated that Nrf2 pathway has a regulatory function in insulin signaling, mitochondrial biogenesis, adipocyte differentiation and liver energy metabolism. So its activation results in increased energy metabolism and suppressed lipid synthesis [98]. In addition, a growing body of evidences supports the role of miRNA as a new important inflammatory mediator in WATby regulating both the adaptive and innate immunity [99].

#### Currently available anti-obesity drugs

In addition to surgery that is only recommended in special conditions, there are some approved drugs for treatment of obesity. However, medical therapy is regarded as an adjunct to lifestyle intervention in obesity.

### Orlistat

Orlistat (Alli®, Xenical®) is a reversible inhibitor of lipase enzymes in the GI tract that can reduce fat absorption from the GI. It breaks down the dietary triglycerides into free fatty acid (FFA) and is also associated with reduction in LDL-C level. It is the only anti-obesity approved drug for use in adolescents. A meta-analysis study showed a 6.5 % weight loss by consumption of Orlistat [100]. Its adverse effects are:steatorrhea, flatulence, kidney stones and rarely liver injury. However its usage is safe for long-term [101].

### Lorcaserin

Lorcaserin (Belviq®) is a serotonin-2C receptor agonist that suppresses appetite and promotes satiety. It can reduce body weight (4.5–5.8 %) through the reduction of energy intake without influencing on energy expenditure. Its adverse effectsincludes: headache, dizziness, fatigue, nausea, dry mouth and constipation in non-diabetics, and hypoglycemia, headache, back pain, cough and fatigue in diabetics and rarely changes in echocardiography, bradycardia, hematological changes and psychiatric disorders. Increased risk of breast cancer in rat has been shown with its consumption. Its long term use has been approved by FDA [101].

### Phentermine/ Topiramateextended-release

Phentermine/Topiramate(PHE/ TPM) extended-release (Qsymia®) is a combination of sympathomimetic and gamma-aminobutyrate agonist that suppresses appetite and promotes satiety. It can reduce body weight (7.8 % by 7.5 mg/46 mg, or 9.8 % by 15 mg/92 mg) and FDA approved it for long-term use. The most common reported adverse effects are: dry mouth, constipation, paresthesia, and dysgeusia. Although no congenital malformations have been reported with its consumption, but it is contraindicated in pregnancy [101].

### Noradrenergic drugs

This group includes Diethylpropin (Tenuate®, Tenuate dospan®), Phentermine (Adipex ®), Benxphetamine (Didrex®), and Phendimertrazine (Bontril®, Prelu-2®) that are approved for short-term use (up to 12 weeks for diethylpropin, and phentermine) by FDA. This group act like norepinephrine and themost prescribed drug among them is Phentermine. Diethylpropin, and Phentermine have potential for drug abuse. Other adverse effects are insomnia, nervousness, dry mouth, tachycardia and blood pressure elevation. The degree of weight loss with Phentermine is dose-dependent [101].

### Anti-obesity drugs in obesity-induced metabolic disorders

Drugs that are suggested for treatment of obese patients with diabetes include: Metformin, Pramlintide, Exenatide, Liraglutide, and Gliflozins. Metformin can reduce hepatic glucose production and suppress hepatic glucose output. A meta-analysis study has showed that short term administration of Metformin in addition to lifestyle modification is acceptably safe and effective among obese adolescents [102]. Pramlintide (Symlin®) is a modified form of amylin that results in weight loss with a mechanism similar to GLP-1 agonists. Exenatide and liraglutide are GLP-1 agonists. Gliflozins such as Dapagliflozin and Canagliflozin are SGLT-2 inhibitors [103].

Drugs that are suggested for treatment of dyslipidemia-associated obesity includes: nicotinic acid and statins [104]. It is shown that combination of omega-3 fatty acids with statins can increase the catabolism of very-low density lipoprotein cholesterol (VLDL-C), intermediate density lipoprotein cholesterol (IDL-C) and LDL-C. Drugs that increase insulin sensitivity such as Metformin or TZDs have no or minimal effects on lipid profile in obesity [104, 105].

### New therapeutic agents

Beloranib (Zafgen®) is a novel injectable anti-obesity agent that can promote intracellular reduction in fat biosynthesis and fat oxidation and lipolysis by inhibition of methionine aminopeptidase-2 (MetAP2). Bupropion/ Naltrexone (Contrave®) is a combination of dopamine and norepinephrine reuptake inhibitor with an opioid receptor antagonist that can reduce appetite, increase energy expenditure, prevent feedback inhibition and further increase pro-opiomelanocortin (POMC) neuronal activity. Amylin, also called islet amyloid polypeptide, acts synergistically with insulin, regulates plasma level of glucose, and mediates appetite suppression. Metreleptin is a leptin analog that suppresses appetite. Exenatide and Liraglutide are GLP-1 agoniststhat increase insulin secretion, decrease glucagon secretion, decrease the gastric secretion and motility, increase satiety, and decrease food intake. TKS1225 and OXY-RPEG are oxyntomodulin (OXM) analogs that reduce weight and appetite [101].

Future directions in medical therapies should be focused on neurochemicals and mediators that regulate food intake, such as agouti-related protein, leptin, neuropeptide Y5, dopamine 3, gut hormones such as GLP-1 agonists, ghrelin antagonist, cholecystokinin agonists, pancreatic hormones; amylin, pancreatic polypeptide analogous, 11-β-hydroxysteroid dehydrogenases inhibitors, diglyceride acyltransferase inhibitors, lipase inhibitors, as well as genetic targets that are associated with obesity [101, 106]. However past and current anti-obesity medications have had serious safety risks especially cardiovascular and psychiatric events, weight gain after the cessation of the drug and potential for drug abuse. Disappointing results after cessation of lifestyle modification or medical therapy indicated the need of other treatment modalities to produce better and long-term benefits, in such cases herbal medicines would be a good alternative [107].

#### Natural products as anti-obesity agents

A systematic review, evaluating the effect of herbal medicines used as anti-obesity in controlled trials, showed their efficacy via weight loss, decrease in body fat, decrease in waist and hip circumferences, and decrease in appetite or food intake with concomitant anti-hyperglycemic, anti-hyperlipidemic, and anti-oxidative effects. Anti-obesity mechanisms for herbal plants includes: reduction in lipid absorption and energy intake, increase in energy expenditure, decrease in differentiation and proliferation of pre-adipocytes, decrease in lipogenesis, and increase in lipolysis [108]. Adverse effects included abdominal bloating and upper respiratory tract infection which was shownonly with alginate-based brown seaweed *Laminaria digitata* [109, 110].

Some examples of plants with anti-obesity effects are: *Cissus quadrangularis*, *Nigella sativa*, oolong tea, black Chinese tea, *Garcinia atroviridis*, *Zingiber officinale*, and supplements containing ephedra and caffeine. *Aracbis bypogaea*, *Allium victorialis*, Pomegranate leaf, *Kocbia scoparia*, *Panax japonicas*, and *Aesculus turbinate* Blume can decrease body weight, liver triglycerides content, liver size, and increase fecal lipid excretion via lipid absorption inhibition [109, 110].

*Cissus quadrangularis*, supplements containing ephedra and caffeine, a natural compound containing capsicum and some lipotropic nutrients, Bofutsushosan, *Irvingia gabonensis* a West African plant, and *Garcinia atroviridis* were resulted in a decrease in body fat. *Zingiber officinale*, *Caralluma fimbriata*, *Cissus quadrangularis*, *Nigella sativa*, and supplements containing ephedra and caffeine are shown to efficiently decrease the waist circumference with/without effect on hip circumference [109, 110].

Effect of supplements containing ephedra and caffeine, *Caralluma fimbriata*, hydroxycitric acid, Fungreek fiber, Epigallocatechin of green tea, and a natural compound containing capsicum and some lipotropic nutrients on food intake have been shown. *Agave tequilana*, Dasylirion spp, Pomegranate leaf, Korean red ginseng, Tree peony root, *Gyeongshang angjeehwan* could decrease the serum lipid levels, decrease appetite, and have an influence on hormonal status [109, 110].

Overall, dietary phytochemicals are classified into polyphenols, terpenoids, organosulfures, and phytosteriols. They can be employed as anti-obesity due to suppression of adipose tissue growth, inhibition of pre-adipocytes differentiation, lipolysis stimulation, and apoptosis induction to reduce adipose tissue mass [111].

### Polyphenols

Resveratrol is an example of a subgroup of polyphenols that is called stilbenes. Resveratrol has antioxidative and anti-obesity effects. Its protective effect against oxidative stress is attributed mostly to its effect on redox enzyme than its moderate ROS-scavenging activity. Resveratrol can promote antioxidant’ enzymes, prevent eNOS uncoupling, and increase expression and activity of eNOS. Its anti-obesity effects are presented via decreasing adipogenesis and viability in maturing preadipocytes, altering fat mass, increasing activity of enzymes related to calorie restriction, increasing lipolysis, inducing apoptosis, reducing lipid accumulation via reduction lipogenesis and proliferation, reducing expression of mediators of inflammatory response such as TNF-α, inhibiting TNF-α-activated NF-κB signaling, modulateing adipokine expression, improving insulin sensitivity and increasing insulin-stimulated glucose uptake [57, 111].

Curcumin is another example that belongs to curcuminoid subgroup of polyphenols. Curcumin can regulate the expression of genes involved in energy metabolism, lipid accumulation, and lipogenesis such as PPARγ, suppress angiogenesis, improve obesity-associated inflammation and obesity-induced metabolic disorders such as insulin resistance, hyperglycemia, and hyperlipidemia, protect from liver injury, prevent LDL-C oxidation, increase adiponectin production, decrease hepatic NF-κB activity and inflammatory markers of liver, reduce body weight gain, adiposity, and microvessel density in adipose tissue, and reduce leptin resistance [111].

### Terpenoids

Terpenoids constitute one of the largest families of natural products. Lycopene is an example of subgroup of terpenoids that is called carotenoid. Lycopene is a powerful antioxidant that can inhibit LDL-C oxidation and lipid peroxidation. Its other biological activities include hormonal and immune system modulation, anti-angiogenesis, inhibition of cell proliferation, induction of apoptosis, reduction of production of pro-inflammatory markers, and attenuation of TNF-α-mediated induction of pro-inflammatory markers [111].

### Organosulfurs

Organosulfur compounds are particularly abundant in *Allium* vegetables such as garlic and onion with bioactive substances including allicin, allixin, and allylsulfides. Anti-obesity effects of garlic and onions include: decreasing the synthesis of cholesterol by hepatocytes through inhibition of hydroxy methylglutaryl coenzyme A (HMG-CoA) reductase, prevention of platelet aggregation, inhibition of activity of the inflammatory enzymes, decreasing the expression of iNOS in macrophages, decreasing the production of inflammatory signaling molecules, decreasing the fat cell number, and consisting antioxidative effects [111].

### Phytosterols

Phytosterols are mainly found in vegetable oils, seeds, and nuts. They can protect against atherosclerosis by decreasing the serum total and LDL-C levelsvia inhibition of intestinal absorption of cholesterol. The potential anti-obesity plant of this group is *Commiphora mukul*. Its effect is due to its active lipid- and glucose-lowering, HDL-C rising and anti-inflammatory agent (guggulsterone). In addition, guggulsterone can improve PPARγ expression and activity and inhibit adipocytes differentiation [111].

### Omega-3 fatty acids

Several clinical trials have explored the effects of oral intake of omega-3 on body weight and body composition. Although, available evidences have shown conflicting results, its beneficial effect on fat mass and waist circumference is reported in several studies [112, 113].

#### Combination therapy of natural products as anti-obesity agents

As complex cell signaling pathways are involved in obesity, it seems that the combination of phytochemicals would result in synergistic effects. A combination of xanthigen (brown marine algae fucoxanthin) plus pomegranate seed oil, *Cissus quadrangularis* with *Irvingia gabonensis*, the combination of *Sambucus nigra* and *Asparagus officinalis*, hydroxycitric acid combined with *Gymnema sylvestre*, resveratrol in combination with genistein and quercetin are shown to have an effective role in the treatment of obesity [109, 110, 114]. Another systematic review showed the beneficial anti-obesity effect for mixtures containing *Ephedra*, *Cissus quadranglularis*, *Ginseng*, bitter melon, and *Zingiber* [115].

### Dyslipidemia

Overall, lipids are insoluble in plasma, however, inorder to be transported to various tissues, they are carried in lipoproteins. Lipoproteins are classified into five major groups; chylomicrones, VLDL-C, IDL-C, LDL-C, and HDL-C. Lipoproteins consist of esterified and un-esterified total cholesterol (TChol), triglycerides (TGs), phospholipids, and protein. However, dyslipidemia is defined as an increase in amount of TChol, TGs, LDL-C, apolipoprotein (apo) B or lipoprotein a (LP a) above 90 % and/ or HDL-C and apo AI under 10 % of general population [116]. The general contribution of hypercholesterolemia to coronary risk is demonstrated clearly, while this association for hypertriglyceridemia remains uncertain. HDL-C in contrast to LDL-C and VLDL-C is anti-atherogenic.

According to WHO statistics, four non-communicable diseases (NCDs) including CVD, cancer, chronic respiratory disease and diabetes were the major causes of mortality in the world in 2008 [117]. Atherosclerosis of the arterial vessel walls is the most important underlying cause of CVD and elevated plasma cholesterol level is a major and primary risk factor of atherosclerotic CVD.

#### Pathophysiology of dyslipidemia

Lipoprotein’s metabolism is performed in exogenous and endogenous pathways. Exogenous pathway can regulate the amount of dietary cholesterol via influencing on its intestinal absorption. Endogenous pathway can transport lipids from the liver by synthesis of VLDL-C in liver which contains a core of TGs and cholesterol esters [116]. Overall, fatty acids are transformed to cholesterol in hepatocytes through HMG- CoA reductase and squalene synthase. Moreover, accumulation of TGs in the liver can be reduced by activating PPARα that results in a decrease in VLDL-C turnover. In addition, VLDL-C can be assembled and secreted through microsomal triglyceride transfer protein (MTP) to form LDL-C. LDL-C can be degraded via LDL receptor (LDLR) called PCSK9 that promotes LDLR catabolism. All of above pathways are happening in hepatocytes. Activation of three subtypes of PPAR (α, β/δ, γ) in adipose tissue, vascular wall, liver, and muscle tissue can result in mitochondrial biogenesis, increase in the oxidation of fatty acids, increase in apo A-I and A-II, nitric oxide, lipoprotein lipase (LPL), and adiponectin level, and decrease in inflammatory cytokines, leptin, and toll-like receptor-4. All of the above events can lead to improve insulin sensitivity, decrease TGs, increase HDL-C, and decrease small dense LDL-C levels [118].

The role of oxidative stress has been shown in physiopathology of dyslipidemia. Superoxide reaction with nitric oxide (NO) may result in peroxynitrite and reducing NO production. Vascular damage, protein oxidation, lipid peroxidase production, and nuclear DNA damage are known as some effects of oxidative stress [119, 120]. Oxidized LDL-C is a chemically modified form of LDL-C that can enter macrophages via unregulated scavenger receptor and form isoprostanes, foam cells and atheromatous plaques [116]. Oxidized LDL-C promotes atherosclerosis via inflammatory and immune responses by stimulation MCP-1, platelet derived growth factor, fibroblast growth factor, TNF-α, interlukin-1, and other cytokines. Some of these cytokines enter circulation and stimulate production of C-reactive protein (CRP) in the liver that is known as a predictor of atherosclerosis [121]. In addition recent studies have demonstrated the important role of miRNA as a regulator of lipoprotein metabolism, oxidative derivatives of lipoprotein, and redox balance [122].

#### Currently available lipid-lowering drugs

It is reasonable that the first step in treatment of hyperlipidemia is lifestyle modifications. However pharmacotherapy is also recommended in selective situations. The available pharmacological medications are discussed bellow.

### HMG-CoAreductase inhibitors

The drugs of this group, so called statin, are effective on cholesterol synthesis and result in reduction of of TChol synthesis and increase in LDL-C uptake via inhibition HMG-CoA reductase. Members of this groupinclude Lovastatin, Pravastatin, Simvastatin, Fluvastatin, Atorvastati, and Rosuvastatin. They have somedifferences in chemical structure, but have a similar target which is reduction of LDL-C. They can decrease LDL-C by 20–55 %, but are not effective in very low baseline levels of LDL-C (<70 mg/dl). Their other benefits include decrease in TChol, and TGs, and 5–10 % increase in HDL-C, improvement of endothelial function, increasing the stability of platelets, decreasing the inflammation and oxidation of lipoprotein, and improvement of blood flow. Their beneficial effectsare shown in patients with specific conditions such as chronic kidney disease (CKD) who do not require dialysis, as well as CKD patients on dialysis. The most serious adverse effects are liver dysfunction and myopathy [121, 123, 124].

Antioxidative effects of statins have been shown to be done through suppression of NADPH oxidase and myeloperoxidase expression, induction of antioxidant’ enzymes and up-regulation of their activity, eNOS uncoupling prevention by up-regulating the GTPCH1 expression, boost of eNOS expression and activity, reduction of circulating oxidized LDL-C, inhibition of LDL-C uptake by macrophages, and reduction of biomarkers of oxidation. In addition, statins exhibit a pleiotropic effect to decrease the ROS formation [120, 125].

### Bile acid sequestrants

The drugs of this group can interrupt the enterohepatic circulation of bile acids by binding to negatively charged bile acids and bile salts in the small intestine. This binding results in increasing the conversation of cholesterol into bile. Increased bile acids secretion results in elevation of TChol oxidation in hepatocytes, increase in LDL-C receptors, increase in LDL-C uptake in liver, decrease in LDL-C serum level, increase in cholesterol synthesis, and promotion of VLDL-C secretion. Some examples are Cholestyramine, Colestipole, and Colesevelam. They can mainly reduce LDL-C levels by 15–25 %. Their adverse effects are TGs elevation, and GI symptoms such as constipation [121].

### Fibric acid derivatives

Fibrate therapy with Fenofibrate or Gemfibrozil can decrease TGs by reducing the production of VLDL-C, and increasing LPL activity through activation of PPARα. Their main reduction effect is on VLDL-C, but have a little beneficial effect on LDL-C. They can decrease TGs by 40 %, increase HDL-C by 10 %, and enhance the formation of large less dense LDL-particles. They can increase the risk of pancreatitis in patients with normal or mildly elevated TGs level, bile stones, and myopathy [121].

### Nicotinic acid

The most inexpensive drug of this group is niacin that can reduce TGs by reduction of VLDL-C production through binding to G protein-coupled receptor (GPR) 109A and inhibition of hormone-sensitive lipase activity. Niacin can directly inhibit hepatic diacylglycerol acyl transferase (DGAT-2) which is the key enzyme in TGs synthesis. This group can reduce TGs by 30–40 %, TChol and LDL-C by 15–30 %, and lipoprotein by 40 %, and increase HDL-C by 15–25 %. Their adverse effects are flashing, hyperglycemia, hepatotoxicity, and gout [121, 126].

### Intestinal cholesterol absorption inhibitor

Zetimibe is the first drug that can prevent absorption of TChol in entrocytes through uncertain mechanism. It can reduce LDL-C levels in a sole administration by 15–20 %, but it is mostly recommended to be consumed in combination with statins which can further reduce LDL-C. However, there are ongoing researches in the assessment of the combination form of Zetimibe with statin [121].

### Omega-3 fatty acids

Lovoza (Omacor®) is a fish oil supplement that contains Eicosapentaenoic acid/Docosahexaenoic acid (EPA/DHA). It is effective on hypertriglyceridemia via reduction of VLDL-C production, but in the U.S.A, it is only recommended in TGs level higher than 500 mg/dl. In patients with TGs level of 200–500 mg/dl, it can reduce TGs by 20 to 50 %, depending on baseline level, decrease VLDL-C by 40 %, minimally increase HDL-C, and increase LDL-C secondary to conversion of VLDL-C to LDL-C. Its most commonly reported adverse effect is an increase in the susceptibility to bleeding while consumed with anti-coagulant drugs [121].

### Lipid-lowering drugs in metabolic disorders

The American Diabetes Association (ADA) recommends HMG-CoA reductase inhibitors, Fibrates, Resins, and intestinal enzymes inhibitors for treatment of hyperlipidemia in diabetic patients [127]. However, an increased risk of developing type 2 diabetes by statin in a meta-analysis study was shown [128]. Some of antidiabetic drugs (insulin and Metformin) can reduce lipid concentration.

### New frontiers in lipid-lowering drugs

By considering the adverse effects of above drugs, development of new medications is needed. The strategies have been focused on production of stronger statins, MTP inhibitors such as Lomitapide, ACAT2 inhibitors, cholesteryl ester transfer protein (CETP) inhibitors such as Torcetrapib, Anacetrapib, Evacetrapib, and Dalcetrapib, antioxidants, squalene synthase inhibitors, preprotein convertase subtilisin kexin-9 inhibitors (PCSK9) such as Regeneron®, selective thyroid hormone mimetics such as Sobetirome as a β-receptor agonist, combination form of PPAR-α and γ agonists (Aleglitazar), PPAR-δ agonists (GW501516), and Apo A-I agonists (CSL-112) [116, 118].

#### Natural products as lipid-lowering agents

Recently, it has been suggested that irregular accumulation of TGs in adipocytes may be a cause of metabolic disorders. This hypothesis has prompted the search for natural products which can reduce the lipid droplet accumulation (LDA). This beneficial effect has been shown in some plant-derived compounds such as berberine, genistein, curcumin, and EGCG. These compounds have been found in *Aristolochia manshuriensis kom*, *Brassica rapa*, *Hibiscus sabdariffa*, *Curcuma longa*, etc. These plants showed their anti-LDA activity via one or more than one way; inhibition of adipogenesis, inhibition of expression of adipogenic factors, inhibition of lipid droplet production, or promotion of lipolysis [129].

### Lipid-lowering plants

Some plants that have shown lipid-lowering effects include: *Satureja* species, *Teucrium* species, *Glycyrrhiza glabra*, black tea, green tea, Fenugreekseeds, and *Rheum ribes* [130–132]. Licorice root extract of *Glycyrrhiza glabra* as a polyphenol, can significantly decreasethe TChol, LDL-C, TGs, and VLDL-C levels via reduction of the susceptibility of LDL-C to oxidation, scavenging free radicals, and inhibition of cholesterol synthesis. Green tea has shown its beneficial effect on TChol and LDL-C via reduction of micellar solubility and intestinal absorption of cholesterol, increase in fecal excretion of fat and cholesterol, reduction of hepatic cholesterol concentration, and up-regulation of LDL-C receptors. Dried leaves of *Satureja khuzestanica*are shown to be effective on TChol, LDL-C, HDL-C, and total antioxidant power via suppression of cholesterol synthesis, reduction of oxidized LDL-C and prevention of malathion-induced activity. Fenugreek seeds, *Trigonella foenum graecum* can decrease TChol, TGs, and VLDL-C levels via several mechanisms that one of them is interaction with bile acids in the GI tract [131].

In more than of 50 % of randomized controlled trials, garlic has been shown to produce its beneficial effects in hyperlipidemia. However, based on a systematic review which was conducted to assess its lipid-lowering effect in controlled trials, garlic could not be recommended tocontrol LDL-C or increase HDL-C [133]. In another systematic review that conducted to assess efficacy and safety of herbal medicines in hyperlipidemia, garlic investigations were more frequent than other natural products, but none of studies with garlic showed its significant effect on hyperlipidemia [131].

### Melatonin

Melatonin can protect LDL-C from oxidation. Other effects include prevention of degenerative disease by protecting DNA, lipids, and proteins from free radical damage. A systematic review of the animal studies and clinical trials showed its beneficial effect on TChol, TGs, LDL-C, lipid peroxidase, TNF-α, body weight, susceptibility of LDL-C to oxidation, HDL-C level, and antioxidative enzymes and glutathion levels. It influences the cholesterol metabolism via modulating the macrophage activity and regulating the secretion of pro-inflammatory cytokines. However, assessment of its safety and efficacy in long-term is required [134, 135].

### Mixture of natural products as lipid-lowering agents

The combination of *Guggul*, *Terminalia belerica*, *Terminalia chebula*, *Embelica officinalis*, ginger, long pepper, and black pepper isshown to significantly decrease TChol, and HDL-C levels versus control group [131]. Other mixtures that have shown beneficial effects on hyperlipidemia includes: extract of theaflovin, green tea catechins, and other tea polyphenols that produced significant decrease in TChol, and LDL-C, and non-significant increase in HDL-C and TGs [131].

### Metabolic syndrome

According to National Cholesterol Education Program Third Adult Treatment Panel (NCEP ATP III), metabolic syndrome (MetS) is characterized by a cluster of three or more features; hyperglycemia, hypertriglyceridemia, low level of HDL-C, and central obesity [136]. The prevalence of MetS has reached epidemic due to increase in its components. Culminate MetS components together could result in 5, and 3 fold increase in the risk of T2DM and CVD, respectively. In addition, MetS is associated with major cardiovascular events and a high mortality rate [137].

#### Pathophysiology of MetS

Most subjects with MetS are obese. Increase in adipose tissue results in elevation of serum FFA and other adipokines. Increase in FFA induces the insulin resistance, elevates the plasma glucose, and in long term impairs the β-cell function. Elevated FFA also lead to increase in hepatic glucose output, worsening of hyperglycemia, elevation of TGs, and attenuation of HDL-C. Obesity is also associated with an increase in blood pressure with an unknown mechanism. Moreover, MetS, as a proinflammatory and prothrombotic state, is associated with an increase in CRP, interleukin-6, and PAI-1 levels that results in insulin resistance predisposition [138, 139]. In addition to genetic susceptibility, and systemic inflammation, oxidative stress has an important role in pathogenesis of MetS, similar to its role in pathogenesis of each component of MetS. Moreover, all MetS components have adverse effects on endothelium, and endothelial dysfunction is prevalent in MetS patients, an event thatis responsiblefor increased risk of T2DM and cardiovascular disease in MetS subjects [140, 141]. So it is expected that subjects with more components of MetS have a higher level of oxidative stress biomarkers.

#### Pharmacological treatment of MetS

MetS is a multifaceted health problem that can not be treated with a single agent. Although, the first step is to have a healthy lifestyle, due to difficulty in initiating and maintaining a healthy lifestyle, a targeted approach for intensive management of all components of MetS is required. Since, in previous sections pharmacotherapy of each component was discussed, here we briefly introduce the remaining medications.

### Currently available anti-MetS drugs

Recommended anti-obesity drugs are: Locaserin, PHE/TPM, and sustained release Bupropion/Naltrexone that their main therapeutic target is central obesity. TZDs, Metformin, GLP-1 agonists, and SGLT-2 inhibitors are recommended as anti-hyperglycemic agents. Statins are recommended as anti-hypertriglyceridemia, while fibrates are recommended in subjects with high levels of TGs, or low level of HDL-C. It is shown that Cilostazol, a selective phosphodiesterase-3 inhibitor, as an anti-thrombotic agent, improves the hypertriglyceridemia, and low level of HDL-C. Finally, angiotensin converting enzyme inhibitors or angiotensin receptor blockersare recommended for hypertension [142]. Most of new target therapies for diabetes and obesity such as miRNA, Nrf2 activators could be useful for MetS treatment.

#### Natural products and MetS

Some of natural antioxidative agents and Nrf2 activators are known to have beneficial effects on MetS. A large number of nutritional supplements or plants such as polyphenols, phytoestrogens, omega-3 fatty acids, chromium, *Cinnamomumcassia, Cinnamomum verum, Artemisia dracunculus, Trigonella foenum-graecum,* and *Vaccinium angustifolium* have been evaluated in this context.

### Polyphenols

Out of polyphenols, flavonoids are commonly studied for prevention and amelioration of MetS associated risk factors. Their beneficial effects on obesity-associated MetS are done via an increasein energy expenditure and fat oxidation and decrease in appetite, adipose tissue inflammation, and glucose absorption. Flavonoids can improve insulin resistance via a decreasein adipose tissue and hepatic inflammation, hepatic oxidative stress attenuation and increase in insulin mediated processes in liver, muscle, and adipose tissue. They can regulate blood pressure via increasing the NO bioavailability and production and decreasing the O_2_^−^ production. Finally, their beneficial effects on hyperlipidemia include an increase in β-oxidation, and decrease in lipogenesisand cholesterol synthesis [143].

### Phytoestrogens

Although animal studies have shown the reduction in adipose tissue and improvement in glucose uptake by intake of soy and phytoestrogens, available human studies do not support above evidences [144].

### Omega-3 fatty acids

The α-linoleic fatty acid, which is found in vegetableoil, is the precursor of some of the omega-3 such as EPA, and DHA. On the other hand, EPA and DHA are found in fish and fish oil and are commonly considered to be marine sourced fatty acids. They have anti-inflammatory and immune regulatory activities via inhibiting the production of inflammatory cytokines and decreasing the leukocyte recruitment and diapedesis. The most important point is the balance between omega-3 and omega-6 concentration which is required for homeostasis maintenance, normal development and mental health. An excess intake of omega-6 promotes pro-inflammatory state, while a high intake of omega-3 has anti-inflammatory effects. An increased intake of omega-3, EPA, and DHA can reduce the risk of cardiovascular disease and improve obesity-associated MetS features such as insulin resistance, hypertension, and dyslipidemia by decreasing the TGs, VLDL-C, BMI and improvement of endothelium vasodilation. Only DHA could increase HDL-C and LDL-C particle size and enhance vasodilatory mechanisms and attenuate constrictor responses in the forearm microcirculation. However, more clinical trials are needed to give us the effective dosage and formula for different pathologies [113].

### Chromium

Trivalent chromium (Cr^3+^) is an essential micronutrient that is found in many dietary sources such as seafood, green vegetables and fruits. Its deficiency is characterized by symptoms similar to MetS, and may result in hyperglycemia, hypertension, central obesity, and decrease in HDL-C. The mechanism of its action is not completely demonstrated. Cr^3+^ is transported in the blood by binding to the transferrin. In the cell, Cr^3+^ is released from transferrin and binds to low-molecular-weight chromium-binding (LMWCr) complex, and becomes a part of the insulin signaling amplification system by enhancing tyrosine kinase activity. Moreover, it increases GLUT4 translocation in muscle tissue of animals. Although, both of above processes result in an increase in insulin sensitivity, effect of chromium on insulin resistance in subjects with diabetes and MetS is controversial. These variations may be related to differences in patient population, dosage, and duration of treatment. But several clinical trials have shown its beneficial effects on lipid metabolism especially after several months of treatment [145].

### Cinnamon

The exact mechanism of action of aqueous cinnamon extract (*Cinnamomum verum*, *Cinnamomum cassia*) is not clear, however, it can increase autophosphorylation, that results in both activation of the insulin receptor andattenuation of tyrosine phosphatase activity. Its beneficial effect on hypertension, TGs and LDL-C also has been reported [146]. It can increase glucose uptake, activate glycogen synthase, and prevent insulin resistance in experimental studies. Ligand-like activity similar to TZDs, and activator receptors of PPAR-γ, and PPAR-α are shown for this substance. So, similar to PPAR-γ agonists, cinnamon can promote differentiation of adipocytes to mature form. Although several clinical trials have shown its beneficial effects on insulin sensitivity and glucose levels in diabetics and healthy people, evidences on MetS are little [145].

### Other plant-derived compounds for treatment of MetS

Some of the selected plant-derived that areeffective on treatment of MetS includes: *Artemisia dracunculus* (increase insulin sensitivity, decrease blood glucose), *Momordica charantia* (increase insulin sensitivity, decrease blood glucose, TGs, and LDL-C), *Trigonella foenum-graecum* (increase insulin sensitivity, decrease blood glucose, TGs, and LDL-C), *Vaccinium angustifolium* (decrease blood pressure, blood glucose, and body weight), *Vitis vinifera* (decrease blood pressure, and LDL-C), *Hoodia gordonii* (decrease body weight), *Crataegus laevigata*, *Crataegus monogyna*, *Crataegus curvisepala*, and *Crataegus tanacetifolia* (decrease blood pressure and LDL-C) [146].

### Osteoporosis

Osteoporosis is a common problem among aging population that results in significant disability and increased morbidity. It is a systemic skeletal disease that characterized by low bone mass, microarchitectural deterioration of bone tissue and enhanced bone fragility and fracture risk. Its causes are different according to sex and age. In postmenopausal women, it is usually the result of accelerated bone turnover secondary to hormonal (estrogen) deficiency. In men and premenopausal women, osteoporos is happens due to vitamin D insufficiency and hyperparathyroidism. In these subjects, osteoporosis is defined as bone mineral density (BMD) ≥2.5 below the average mean peak bone mass of young adults that measured by a dual-energy x-ray absorptiometry (DEXA) [147]. The DEXA scan generally measures the bone mineral content of either the lumbar spine or proximal femur. The low bone mass in children and adolescents (<20 years of age) has been defined by a Z-score below -2. International Osteoporosis Foundation (IOF) estimated that currently there are more than 200 million osteoporotic people in the world. Its prevalence in women is more than men (34 % versus 17 %). A 1.4–2.3 fold increase in risk of future fracture in patients with past history of fracture compared to individuals without prior history has been reported [148].

#### Pathophysiology of osteoporosis

Bone is made up of two forms of bone; trabecular bone that consists of spongy marrow and finds at the end of long bones and cortical bone that is denser and forms the outliers of bone. The creation of bone is regulated by the balanced modeling and remodeling processes which osteoblasts (bone-forming cells) and osteoclasts (bone-reabsorbing cells) are responsible for these processes. Osteoporosis happens when an imbalance between osteoblasts and osteoclasts activities develops. The spine and hip bones are at greater risk of fracture due to high rate of trabecular bones in these sites. Multiple hormones are effective in bone development with three hormones having more significant role; parathyroid hormone (PTH) that speeds up bone breakdown, increases production of activated vitamin D and increases intestinal absorption of calcium, calcitonin that conserves calcium, and blocks the PTH effects, and finally vitamin D [149].

In response to microdamage, osteocytes provide resorption signals to osteoclasts. Osteoclasts’ function and differentiation are regulated by receptor activator of NF-κB RANK ligand (RANK-RANKL) signaling pathway, an immunological pathway which is negatively regulated by osteoprotegerin (OPG). PTH binds to the PTH receptor on osteoblasts and by up-regulating RANKL and down-regulating OPG expression, indirectly stimulates osteoclasts’ activity. Binding of calcitonin to its receptorresults in reversible inhibition of osteoclasts’ function. Estrogen inserts its positive effect on bone through its receptor on osteoblasts and osteoclasts. After resorbing osteoclasts, cathepsin k that is required to degrade collagen, is secreted. Osteoblasts’ function and differentiation is regulated by endogenous inhibitors such as sclerostin that is effective on wingless-type and integrase 1 (Wnt) signaling pathway through lipoprotein-related protein 5/6 (LRP5/6) and Frizzled co-receptors [149].

In addition, ROS play a critical role in the pathogenesis of osteoporosis. Accumulation of ROS is associated with loss of bone mass, decrease in bone formation, decrease inosteoblasts numbers and function, and decrease in antioxidant defense responses. So, it is expected that antioxidant supplementation can decrease oxidative stress parameters and bone resorption markers. Moreover an inverse relationship between adipocytes and osteoblasts in the bone marrow has been shown. PPARγ is a master regulator of adipogenesis that negatively regulates bone formation in bone marrow. Similarly, bone is involved in the development of insulin sensitivity. On the other word, there is a network that links osteoporosis to obesity and diabetes mellitus [114, 150, 151].

#### Current therapeutic options for osteoporosis

According to validated guidelines dietary supplementation, bisphosphonates, hormones, and biologic agents could be used in certain conditions.

### Dietary supplementation

Main sources of calcium are dairy products, green leafy vegetables, nuts, seafood and fortified foods. Calcium acts on bone mineralization and metabolism [152]. Its most common adverse effect is gastrointestinal problems. Other adverse effects are: renal calculi, higher hip fracture rates with high calcium intakes and cardiovascular disease especially myocardial infarction [153]. Vitamin D is found in oily fish, eggs and fortified foods. It can also be naturally synthesized through sunlight exposure. Vitamin D can stimulate bone mineralization and increase intestinal absorption of calcium and phosphorus [152]. An increased risk of falls and fractures in elderly women with annual oral dose of 500,000 IU of vitamin D has been reported. Of note is a U-shaped relationship between serum 25-hydroxyvitamin D levels and mortality [153]. Antioxidant effects including an improvement in the cellular GSH level and decrease in ROS and pro-inflammatory cytokines have been found for vitamin D [154]. A meta-analysis showed that vitamin D and calcium may promote β-cell function and insulin sensitivity [155].

### Bisphosphonates

Bisphosphonates are the most common used drugs for treatment of osteoporosis. This group includes: Alendronate (Fosamax®), Rrisendronate (Actonel®), Etidronate (Didronel®), Ibandronate (Boniva®), and Zelodronate (Zometa®, Reclast®). They can increase bone mineral density, reduce the risk of osteoporotic fracture by inactivating osteoclasts bone resorption, and increase osteoclasts apoptosis. Common reported adverse effects are: dyspepsia and esophagitis. Rare side effects include: osteonecrosis of jaw, atypical subtrochanteric femoral fractures, and increase incidence of atrial fibrillation [152, 153].

### Hormones

Tetrapeptide (Forteo®) is a recombinant human parathyroid 1–34 (a PTH analogue) that has an osteoanabolic effect in intermittent injection. It can activate osteoblasts to create new bone, improve BMD, and prevent nonvertebral fractures. It is approved only for usage in women with high risk of fracture and BMD < -3.0 T score, howeverthere is insufficient evidence on its safety for long term usage [156–158]. Its reported adverse effects are: dizziness, leg cramp and local skin reaction at the site of injection. Calcitonin is a natural polypeptide hormone that is secreted by C-cells of the thyroid. It can inhibit bone resorption lesser than other anti-resorptive drugs, increase osteoblasts activity, help to regulate calcium metabolism during physiological stress such as pregnancy, and reduce nonvertebral fractures. Estrogens (Tamoxifen, Raloxifene) can decrease bone resorption by binding to osteoclasts receptors, stimulate the release of mediators for blocking osteoclasts activity, stimulate osteoblasts, and protect against bone loss at all skeletal sites. Estrogens may be able to protect againstoxidative stress. Long term usage was associated with increased thrombo-embolic events and strokes. Estrogen-progestin therapy can also increase the risk of coronary heart diseases and breast cancer [156, 157]. Theoretically, androgen therapy could be anabolic for bone and muscle in older men with osteoporosis, sarcopenia and falls, but a meta-analysis concluded that prevention of bone loss or increase BMD was inconclusive for femoral neck occurrence [159]. However, in guidelines adding an anti-osteoporotic drug to androgen replacement therapy for men with idiopathic hypogonadism at high fracture risk is suggested. Safety of androgens is shown in cardiovascular system, especially in frail older men. They also have little detrimental effects on prostate tissue [160].

### Biologic agents

Denosumab (Prolia) is a monoclonal antibody that by interacting with RANK can prevent RANKL and fracture at multiple sites. In addition it can mimic OPG effect and inhibit bone resorption. Significant reduction in risk of vertebral fracture (68 %), hip fracture (40 %), and nonvertebral fracture (20 %) has been reported with Densumab consumption. Its long term safety is not clear [156]. Its reported side effects are osteonecrosis of the jaw and atypical femoral fracture [158].

### New drugs

Strontium ranelate (Protelos®) can increase BMD and reduce the risk of vertebral and nonvertebral fractures by increasing functional osteoblasts and simultaneously decreasing osteoclasts through calcium sensing receptor (CaSR). This drug is not approved by the FDA, but is licensed for the prevention of vertebral and nonvertebral fracture in subjects with failed or contraindicated bisphosphonate therapy in the Europe. Strontium ranelate causes hypersensitivity, and vascular events [157, 158].

AMG785 and AMG167 are humanized monoclonal antibodies against sclerostin that have not yet completed all phases of clinical trials. Sclerostin is a negative regulator of bone formation that by interaction between Wnt ligand and LRP5/6 co-receptor on osteoblasts can inhibit Wnt signaling and result in reduction of BMD [157]. Dickkopf 1 is another inhibitor of Wnt signaling and Dickkopf 1 antagoinst can increase bone formation. Other drugs include: frizzled-related protein inhibitor, glycogen synthase kinase 3 (GSK-3) inhibitors; lithium, 603281-31-8, 6-bromoindirubin-3^’^-oxime, and AR28 [161].

Odanacatib is a selective, reversible inhibitor of cathepsin K that is reached Phase III clinical trials in postmenopausal women and has shown a positive effect on BMD. Cathepsin K is a lysosomal cystein protease that released by osteoclasts during bone resorption [157].

#### Natural products for treatment of osteoporosis

By considering reports of adverse effects of pharmacotherapy (estrogens, bisphosphonates) in the management of osteoporosis, there is an increasing demand for complementary and alternative medicine. The beneficial effects of antioxidants in bone health and osteoporosis are demonstrated both epidemiologically and through clinical intervention. The medicinal plants are studied widely in recent years and have shown their antioxidative effects on osteoporosis [72]. Some of the antioxidants that areeffective in the oxidative stress prevention in osteoblasts include: green tea, flavonoids from *Parsimmon*, *Panaxnotaginseng saponin*, crysoeriol from *Surya cilliata* leaves, quercetin, *Cathamus tinctorium* flower extract, and *Atractylodes japonica* root extract. Other antioxidants that were shown to affect osteoclasts include polyphenol extracts from dried plums, curcumin, *Salvia miltorrhiza*, and coffeediterpene Kahweol [150]. Other natural products are including omega-3 fatty acids, and phytoestrogens. Soy isoflavones, black cohosh, *Fructus linguistri lucidi*, and *Cissus quadrangularis* have been employed in several clinical trials that used herbal products implicated in maintenance of bone health [161].

### Lycopene

Lycopene is a carotinoid lipid-soluble antioxidant with singlet oxygen-quenching ability 2-fold of ß-carotene and 10- fold of α-tocopherol. Its sources are tomato, watermelon, pink guavas, and pink grape fruit. Since lycopene is a lipid-soluble compound the presence of small amounts of lipids is necessary to enhance its absorption. The most health benefits of lycopene may be due to its potent antioxidant property. Lycopene shows a stimulatory effect on cell proliferation of osteoblasts and alkaline phosphatase activity. It also inhibits the formation of ROS secreting osteoclasts, enhances BMD, decreases the risk of fragility fracture related to osteoporosis, and attenuates N-telopeptide (NTx), a marker of bone resorption [150].

### Polyphenols

Polyphenols are water-soluble antioxidants that naturally are found in plants. Tea, the dried leaves of *Camellia sinensis* is a popular beverage consumed all over the world. Tea polyphenols increase bone formation, inhibit bone resorption, and increase bone strength through antioxidant or anti-inflammatory pathways. The most commonly studied polyphenol, which is abundant in green tea, is EGCG. EGCG increases the formation ofmineralized bone nodules by human osteoblast-like cells, inhibits the expression of matrix metalloproteinase 9 (MMP-9) and the formation of osteoclasts, inhibits thyroid hormone-stimulated osteocalcin synthesis in osteoblasts, and inhibits ratosteoclast formation and differentiation. Polyphenolscan affectosteoblast functions throughsome cellular signaling such as mitogen-activated protein kinase (MAPK), bone morphogenetic protein (BMP), and OPG/RANKL [150]. The beneficial effects of other polyphenols include: attenuation of the detrimental effects of TNF-α on osteoblast function by dried plum, enhancement of osteoblastogenesis and inhibition of adipogenesis and also effect on the differentiation of stem cells derived from bone marrow in usage oleuropein.

### Omega-3 fatty acids

The sources of omega-3 fatty acids are fish, eggs, walnuts and flax seed. They have not only antioxidant function, but also up-regulate intestinal absorption of calcium. An increase in hip, lumbar spine and total body BMD, decrease in bone resorption, and reduction in bone turnover havebeen shown by omega-3 [161]. In addition, they can modulate prostaglandin production and change the production of interleukin-1β, interleukin-6, and TNF-α [162].

### Phytoesterogens

Phytoestrogens includeisoflavones, lignans, flavonoids, stilbenes (resveratrol), and coumestans. They are found in some plants such as *Humulus lupulus*, *Vitex agnus-castus*, *Dioscorea villosa*, *Linum* usitatissimum, *Maritime* pine bark extract, *Cruciferous* vegetables, and Lepidium *meyenii* [163]. It is believed that they bind to estrogen receptors, act as a potent antioxidants and inhibit aromatase and cytochrome P450. Aromatase converts androgens into estrogens and in high level this enzymecanincrease the risk of breast, adrenal and prostate cancers [164]. Two systematic review and meta-analysis studies of randomized controlled trials by ingestion of soy isoflavones in menopausal women revealed a significant improvement in lumbar spine BMD, significant decrease in urine deoxypyridinoline as a bone resorption marker, improvement in bone strength and decrease in the risk of fracture, but did not affect the serum alkaline phosphatase and osteocalcin as bone formation markers [165, 166]. In addition, a synergistic effect of vitamin D and phytoestrogens on bone, but with unclear mechanism, isshown.

Despite inconsistency in results of clinical trials by isoflavones, due to heterogeneity of the trials and suboptimal reporting, there are many unanswered questions regarding which metabolite and at what dose would provide the best health benefits. However, there will always be a need for non-hormonal alteration for relief of menopausal symptoms and phytoestrogens might represent the best alternative to estrogen therapy due to lack of estrogen-induced tumogenecity.

### Combination therapy of natural products as anti-osteoporotic agents

The major components that have been used in herbal combinations for prevention of aging are polyphenols. The greens + ™, a blendof several polyphenolsincluding quercetin, apigenin and luteolin have shown a good source of polyphenols with stimulatory effect on mineralized bone nodule formation in humanosteoblast cells in a dose- and time-dependent manner. It is more effective than epicatechin alone that found in green tea [150]. Another mixture is omega-3 fatty acid combinations that have shown beneficial effects on postmenopausal osteoporotic women [167]. Resveratrol in combination with vitamin D is shown to prevent the weight gain and bone loss in a postmenopausal rat model [114].

## Conclusions

Although the plant derived compounds’ effects, as therapeutic tools, are very smaller than synthetic drugs, the risk of their adverse effects seems to be very rare. Since metabolic disorders are multifactorial, it seems that we need poly-herbal medications for obtaining proper response. However even though there is an increasing interest in herbal therapy, a gap in scientific evidence of plant-derived therapeutic benefits exist. One of the reasons may be slow rate of translation of animal studies’ findings into human clinical trials. Moreover further researches to determine the most effective plant-derived metabolites and their cellular mechanism in order to set priorities for well-designed animal and clinical trials, and also more studies with strong scientific evidences such as a systematic review of controlled studies in poly-herbal medications and drug-plant combinations are needed.

## Abbreviations

ACEI: Angiotensin-converting enzyme inhibitors
ADA: American Diabetes Association
AGEs: Glycation end products
AMPK: AMP-activated protein kinase
Angptl: 2 Angiopoietin-like protein 2
Apo: Apolipoprotein
ARE: Antioxidant response element
BAT: Brown adipose tissue
BMD: Bone mineral density
BMI: Body Mass Index
BMP: Bone morphogenetic protein
CaSR: Calcium sensing receptor
CAT: Catalaze
CETP: Cholesteryl ester transfer protein
CKD: Chronic kidney disease
Cr^3+^: Trivalent chromium
CRP: C-reactive protein
COX-2: Cyclooxygenase-2
CVD: Cardiovascular diseases
DDP-4: Dipeptidyl peptidase-4
DEXA: Dual-energy x-ray Absorptiometry
DGAT-2: Diacylglycerol acyl transferase
EGCG: Epigallocatechin-3-gallate
EPA/DHA: Eicosapentaenoic acid/Docosahexaenoic acid
EPHX2: Epoxide hydrolase
ERK-1/2: Extracellular signal-related kinase-1/2
FDA: Food and Drug Administration
FFA: Free fatty acid
FGF21: Fibroblast growth factor 21
GI: Gasterointestinal
GLP-1: Glucagon-like peptide-1
GPR G: protein-coupled receptor
GSK-3: Glycogen synthase kinase 3
H_2_O_2_: Hhydrogen peroxide
HDL-C: High density lipoprotein cholesterol
HMG-CoA reductase: Hydroxy methylglutaryl coenzyme A reductase
HOMA index: Homeostatic model assessment of insulin resistance
IDF: International Diabetes Federation
IDL-C: Intermedate density lipoprotein cholesterol
IL-1Ra: Interleukin-1 receptor antagonist
INOS: Inducible nitric oxide synthase
JNK1: C-Jun N-terminal kinase
Keap1: Kelch-like ECH-associated protein 1
LDA: Lipid droplet accumulation
LDL-C: Low density lipoprotein cholesterol
LDLR: LDL receptor
LP a: Lipoprotein a
LMWCr: Low-molecular-weight chromium-binding
LPL: Lipoprotein lipase
LRP5/6: Lipoprotein-related protein 5/6
MAPK: Mitogen-activated protein kinase
MCP-1: Monocyte chemoattractant protein-1
MetAP2: Methionine aminopeptidase-2
MetS: Metabolic syndrome
MiRNA: MicroRNA
MMP-9: Matrix metalloproteinase 9
MTP: Microsomal triglyceride transfer protein
NCDs: Non-communicable diseases
NCEP ATP III: National Cholesterol Education Program Third Adult Treatment Panel
NF-κB: Nuclear factor-κB
NO: Nitric oxide
Nox family: NADPH oxidase
NPH: Neutral Protamine Hagedorn
Nrf2: Nuclear factor-erythroid 2-related factor 2
NTx: N-telopeptide
OPG: Osteoprotegerin
OXM: Oxyntomodulin
P38-MAPK: P38 mitogen-activated protein kinase
PAI-1: Plasminogen activator inhibitor-1
PCSK9: Preprotein convertase subtilisin kexin-9 inhibitors
PDEIs: Phosphodiesterase inhibitors
PGI_2_: Prostaglandin I_2_
PHE/ TPM: Phentermine/Topiramate
PKC: Protein kinase C
POMC: Pro-opiomelanocortin
PPARγ: Peroxisome proliferator-activated receptor γ
PTH: Parathyroid hormone
RNS: Reactive nitrogen species
ROS: Reactive oxygen species
Sfrp-5: Secreted frizzled-related protein 5
SGLT2: Sodium-glucose transporter 2
SOD: Superoxide dismutase
SR-A: Scavenger receptor-A
STAT-JAK: Janus kinase-signal transducer and activator of transcription
T2DM: Type 2 diabetes mellitus
TBARs: Thiobarbituric acid reactants
TChol: Total cholesterol
TGs: Triglycerides
TNF-α: Tumor necrosis factor-α
TZDs: Thiazolidinediones
VCAM-1: Vascular cell adhesion molecule-1
VLDL-C: Very low density lipoprotein cholesterol
WAT: White adipose tissue
WHO: World Health Organization
Wnt: Wingless-type and integrase 1
